# Do Benzodiazepines Impair Motor and Nonmotor Symptoms in a Sample of Parkinson’s Disease Patients?

**DOI:** 10.7759/cureus.13220

**Published:** 2021-02-08

**Authors:** Wendy Gaztanaga, Marina Sarno, Jason Margolesky, Corneliu Luca, Carlos Singer, Henry Moore, Jonathan Jagid, Bonnie Levin

**Affiliations:** 1 Neurology, University of Miami, Miami, USA; 2 Neurological Surgery, University of Miami, Miami, USA

**Keywords:** benzodiazepines, parkinson’s disease, motor, cognitive, mood

## Abstract

Background

Anxiety and sleep disturbances are prevalent in Parkinson’s disease (PD). Benzodiazepines (BZDs) are commonly used to treat these symptoms; however, they are associated with unfavorable side effects such as falls and cognitive slowing in the general non-PD population. Examining the effects of BZDs in PD is imperative as these medications could pose an increased risk to PD patients who are already vulnerable to falls and cognitive deficits.

Methods

Eighty-four patients diagnosed with idiopathic PD, of which 60% were Hispanic, underwent clinical evaluations including the Unified Parkinson’s Disease Rating Scale (UPDRS) and comprehensive neuropsychological testing examining global cognition, language, visuospatial skills, memory, executive function, mood, and sleep quality. Thirty-six patients taking BZDs (BZD+) were compared to forty-eight patients not using any BZDs (BZD-) employing appropriate statistical tests depending on the measures’ characteristics.

Results

BZD+ PD patients performed below the BZD- group on short-term memory but not on delayed recall, and performed better on a measure of visuospatial judgment. The BZD+ group endorsed more symptoms of anxiety and depression as well as poorer sleep quality. No significant differences were noted on other measures of cognition or motor function.

Conclusion

PD patients taking BZDs may experience select changes in cognition and mood. These changes are isolated and mild, and suggest that for some patients, BZDs may be a viable pharmacologic intervention that does not alter cognitive and motor function compared to those not taking these medications.

## Introduction

Non-motor symptoms such as anxiety and sleep disturbances are highly prevalent in Parkinson’s disease (PD) [1, 2]. Studies have found that 20-50% of PD patients have anxiety and as many as 90% of PD patients report experiencing sleep disturbances [1]. Sedatives such as benzodiazepines (BZD) are commonly used to treat these symptoms in PD patients [2, 3].

In the general population, benzodiazepines (BZDs) are generally thought to increase the risk of falls and cognitive impairment, particularly in the elderly [4]. Acute administration of BZDs has been known to produce sedation, drowsiness, psychomotor slowing, anterograde amnesia, and difficulties learning new material [5]. Similarly, long-term intake of BZDs has been associated with cognitive decline [6]. A meta-analysis by Barker and colleagues investigated the long-term effects of BZDs on 12 cognitive domains (i.e., sensory processing, psychomotor speed, nonverbal memory, visuospatial, processing speed, problem solving, attention, verbal memory, general intelligence, motor control, working memory, and verbal reasoning) and found significant cognitive decline on all 12 domains in long-term BZD users [6]. Another study examining 3,777 community-dwelling elderly people found that individuals who had used BZDs at least once in their lifetime were at a significantly increased risk for developing dementia [7]. Other studies have also found that elderly individuals taking BZD were at an increased risk for hip fractures [8]. The American Geriatric Society has therefore recommended avoiding BZDs in adults 65 years of age or older [9]. 

The side effect profiles for these drugs in the general population may suggest their unsuitability for PD patients who are already vulnerable to developing impaired cognition and gait dysfunction [10]. However, given the prevalence of anxiety and sleep disturbances in PD and the lack of alternative treatment options, these drugs are nonetheless frequently prescribed [2, 3, 11]. Therefore, it is important to carefully assess and document the effects of BZDs in PD in order to provide better clinical care for these patients. 

There have been few published studies on the response of PD patients to BZDs and most of the research in this area has focused on the therapeutic effects of the drugs, rather than their side effect profile. Casacchia and colleagues in 1975 found that bromazepam, a long-acting BZD, improved emotional and somatic symptoms of anxiety in PD patients [12]. Several published case reports also describe the beneficial effect of clonazepam in treating intractable anxiety in PD [13]. BZDs are thought to be especially useful in PD patients with anxiety and comorbid sleep disturbances [2]. The one study that addressed possible side effects of these medications in PD examined 647 PD patients and found that sedative use was associated with the presence of falls while controlling for disease progression [14]. Cognitive adverse events from sedative use remain understudied in PD patients.

Therefore, the primary aim of this study was to examine the effects of BZD use on motor and non-motor functions in PD. Given that PD patients are, overall, at increased risk of falls and poorer cognitive outcomes compared to the general population, we examined whether PD patients using BZDs would exhibit more impaired cognition and increased risk of falling compared to PD patients not using these sedatives. We further examined the influence of these medications on symptoms of overall mood disturbances and impaired sleep, two common complaints among PD patients who are prescribed sedatives.

## Materials and methods

Participants

Participants were recruited from the Movement Disorders Clinic at the University of Miami Miller School of Medicine. All participants underwent neurologic examinations by a movement disorder specialist, and comprehensive neuropsychological testing by a neuropsychologist as part of a comprehensive evaluation for deep brain stimulation (DBS) surgery. Subjects met diagnostic criteria for idiopathic PD based on UK PD Brain Bank Criteria [15]. Exclusion criteria included (1) a previously established diagnosis of a comorbid movement or neurodegenerative disorder other than PD, (2) moderate to severe dementia as defined by an Mini-Mental State Examination (MMSE) score lower than 25, (3) history of substance abuse, and (4) history of uncontrolled mental illness such as schizophrenia, bipolar disorder, obsessive-compulsive disorder (OCD), and post-traumatic stress disorder (PTSD). This information was gathered during clinical interviews with a neurologist and/or neuropsychologist and corroborated through an extensive review of patients’ electronic medical records. A sample of 102 patients was identified, with 18 patients excluded (i.e., 13 were excluded because their MMSE score was lower than 25, two were excluded due to a comorbid diagnosis of essential tremor, and three were excluded due to a history of OCD, PTSD, and/or psychosis). A total of 84 participants met inclusion/exclusion criteria.

Participants were categorized into two groups based on their BZD use at the time of evaluation: BZD+ (n= 36) and BZD- (n= 48). The groups were defined by information gathered from the neurologist, neuropsychologist, and corroborated data from the electronic medical records regarding their sedative use.

Procedures and data collection

All study procedures were approved by the University of Miami Miller School of Medicine institutional review board. Each patient was evaluated by a movement disorder specialist in the Department of Neurology and a neuropsychologist in the Division of Neuropsychology at the University of Miami in their primary language [English (n= 49) or Spanish (n= 37)].

Measures

The neuropsychological battery was comprised of tests known to be clinically sensitive to cognition in PD [16]. The battery included: the Mini-Mental State Examination (MMSE) as a measure of global cognition [17]; Controlled Oral Word-Association Test (COWAT) (FAS/PTM) to assess language [18]; Judgment of Line Orientation Test (JLO, 15-item version) to measure visuospatial skills [19]; California Verbal Learning Test, Second Edition Short Delay Free Recall (CVLT-2 SDFR) to assess short-term memory recall [20]; CVLT-2 Long Delay Free Recall (CVLT-2 LDFR) to test for long-term memory recall [20]; CVLT-2 Trials 1-5 (CVLT-2 1-5) to assess for learning [20]; and oral Trail Making Test Part B (Trails B) to assess executive functions [21]. Tests were administered in the primary language of the patient. Normative T-scores, adjusted for age and education, were used when analyzing these tests with the exception of the MMSE, which used raw scores.

The Unified Parkinson’s Disease Rating Scale (UPDRS) was used as a motor scale administered while the patient was off PD medications [22]. Reported falls as a subjective symptom (yes/no) was also included in the analysis.

Mood self-report measures included the Beck Depression Inventory - Second Edition (BDI-II) [23] and Beck Anxiety Inventory (BAI) [24] to assess depression and anxiety, respectively. Sleep quality was assessed in a subset of 42 patients, 17 BZD+, using the Pittsburg Sleep Quality Index (PSQI) [25].

Statistical analyses

Clinically relevant variables such as education level, gender, ethnicity, PD duration, and age of PD onset were examined based on their well-established relationships with cognitive outcomes in the literature [26]. As age-corrected normative scores were used as the primary outcome measures in the study, it was not considered necessary to statistically control for age in subsequent statistical models.

Variables that had a normal distribution were analyzed using student t-tests to compare performance on neuropsychological tests, motor scale, and mood and sleep self-report measures between BZD+ and BZD- groups. Variables that did not have a normal distribution such as the MMSE, COWAT, Trails B, BDI-II, BAI, PSQI, and UPDRS, were analyzed using a Mann-Whitney U test instead. A Chi-square test was done to assess whether patients on sedatives reported falling more often than their counterparts not taking sedatives.

## Results

Descriptive statistics

The study sample consisted of 84 participants, 60 females and 24 males, with a mean age of 64.5 ± 9.3 years and an average of 13.6 ± 3.7 years of education. Sixty percent (n= 50) of the sample was Hispanic and 43% of the sample (n= 36) were Spanish-speaking monolingual. On average, the participants were diagnosed with PD at 57.0 ± 11.3 years of age, with a PD duration of 9.3 ± 4.2 years.

The BZD+ group consisted of 36 participants currently taking BZDs (e.g., alprazolam, clonazepam, lorazepam, diazepam, and temazepam.) Within this group, 30 patients were taking one BZD, three were taking a combination of two BZDs, one was taking one BZD plus zolpidem, and two were taking two BZDs plus zolpidem. Dosages ranged from 0.5 mg to 5.2 mg maximum per day (expressed as clonazepam equivalent) and were prescribed on an “as needed” basis [27]. Patients were taking these drugs for an average of 2.09 ± 1.981 years, with a range of two months to six years. Meanwhile, the BZD- group consisted of 48 patients who reported never receiving treatment using BZDs. All demographic information and disease characteristics for these groups are shown in Table 1. Overall, there were no clinically or statistically significant differences between the groups on age, years of education, gender, ethnicity, age of PD onset, or years of disease duration.

**Table 1:** Demographic information for both groups and the overall sample. Data are means ± SD or frequencies, as appropriate. BZD: benzodiazepines

	BZD (+) n = 36	BZD (-) n = 48	Overall Sample n = 84
Age, years	63.7 ± 10.1	65.1 ± 8.7	64.8 ± 9.2
Education, years	14.6 ± 3.8	12.9 ± 3.4	13.6 ± 3.7
Gender (male/female), n	10/26	14/34	24/60
Ethnicity (Hispanic/Nonhispanic/Unknown), n	17/18/1	33/14/1	50/32/2
Disease duration, years	8.4 ± 4.2	9.9 ± 4.1	9.3 ± 4.1
Age of onset, years	58.2 ± 13.3	56.2 ± 9.4	57.0 ± 11.3

Group comparisons

**Figure 1:**
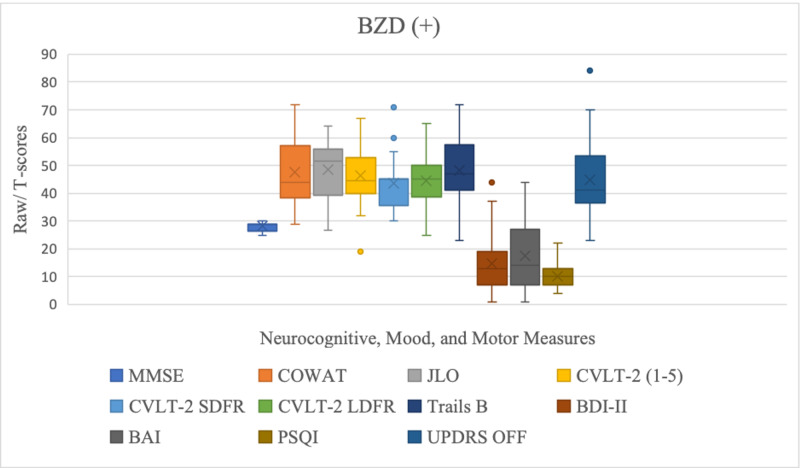
Neurocognitive, mood, and motor scores for the BZD+ group. BZD: benzodiazepines; MMSE: Mini Mental State Examination; COWAT: Controlled Oral Word-Association Test; JLO: Judgment of Line Orientation; CVLT-2 SDRF: California Verbal Learning Test, Second Edition Short Delay Free Recall; CVLT-2 LDFR: California Verbal Learning Test, Second Edition Long Delay Free Recall; Trails B: Trail Making Test Part B; BDI-II: Beck Depression Inventory - Second Edition ; BAI: Beck Anxiety Inventory; PSQI: Pittsburg Sleep Quality Index; UPDRS: Unified Parkinson’s Disease Rating Scale

**Figure 2:**
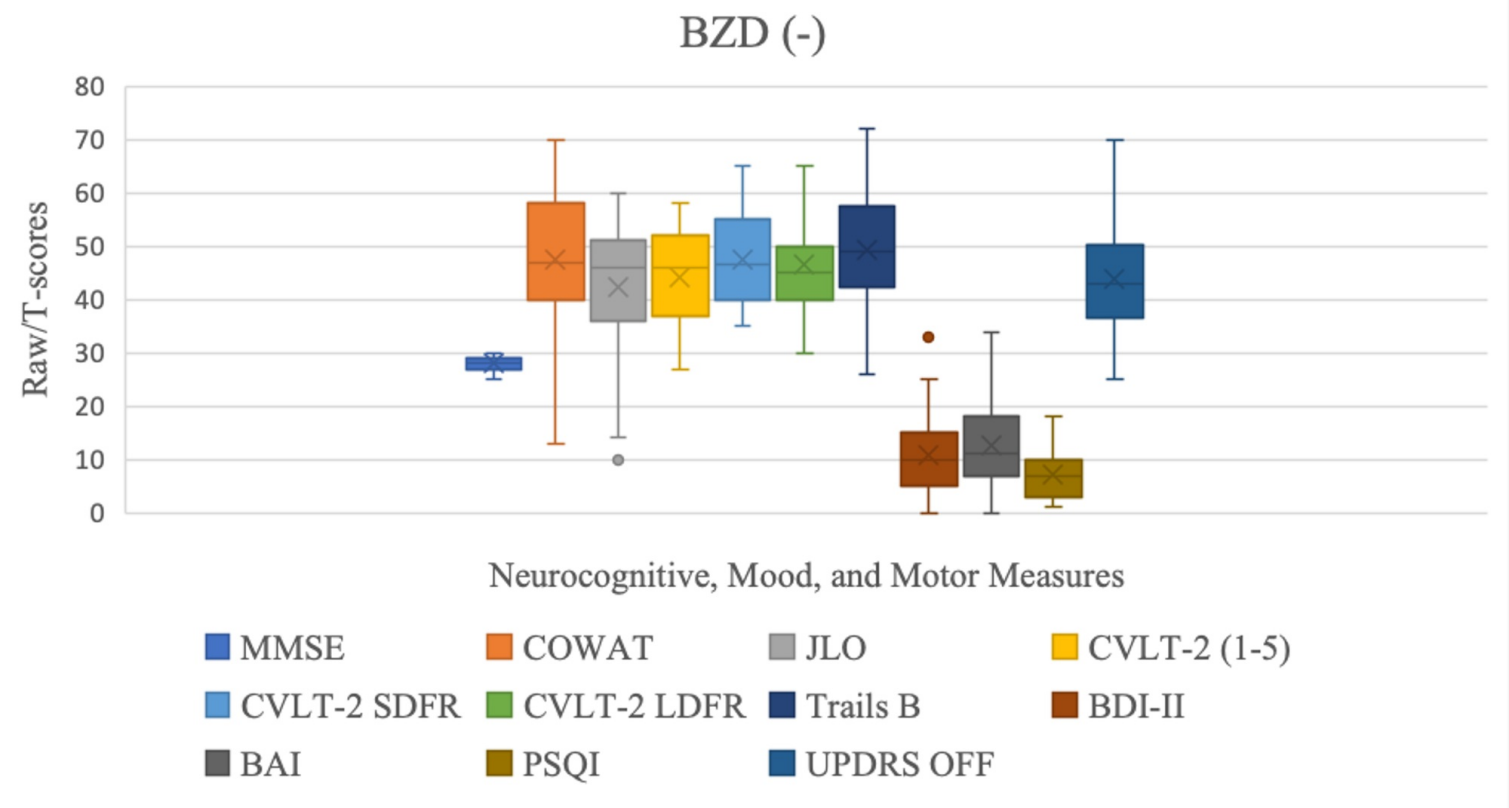
Neurocognitive, mood, and motor scores for the BZD- group. BZD: benzodiazepines; MMSE: Mini Mental State Examination; COWAT: Controlled Oral Word-Association Test; JLO: Judgment of Line Orientation; CVLT-2 SDRF: California Verbal Learning Test, Second Edition Short Delay Free Recall; CVLT-2 LDFR: California Verbal Learning Test, Second Edition Long Delay Free Recall; Trails B: Trail Making Test Part B; BDI-II: Beck Depression Inventory - Second Edition ; BAI: Beck Anxiety Inventory; PSQI: Pittsburg Sleep Quality Index; UPDRS: Unified Parkinson’s Disease Rating Scale

Amongst the measures tested, there were statistically significant differences between the two groups on measures of short-term memory (CVLT-2 SDFR: BZD+: 45 (36.3-45), BZD-: 46.7 (40-55), p= 0.03) where the BZD+ group performed worse than BZD- group; visuospatial ability (JLO: BZD+: 51.7 (41.1-55.8), BZD-: 45 (37.1-50.2), p= 0.016) where the BZD+ group outperformed the BZD- group; as well as depression (BDI-II: BZD+:13 (7.5-18), BZD(-): 10 (5-15), p= 0.046); anxiety (BAI: BZD+:14 (7.5-27), BZD-: 11 (7-18), p= 0.039); and sleep quality (PSQI: BZD+:10 (7-13), BZD:7 (3.5-9.5), p= 0.039) where the BZD+ group reported worse symptoms of depression, anxiety, and sleep quality, as depicted on Table 2 and Figures 1, 2.

**Table 2:** Neurocognitive measures, mood and sleep self-reports, and motor scores for both groups. Data are reported as median (interquartile range) or frequencies, as appropriate. BZD: benzodiazepines; MMSE: Mini Mental State Examination; COWAT: Controlled Oral Word-Association Test; JLO: Judgment of Line Orientation; CVLT-2 SDRF: California Verbal Learning Test, Second Edition Short Delay Free Recall; CVLT-2 LDFR: California Verbal Learning Test, Second Edition Long Delay Free Recall; Trails B: Trail Making Test Part B; BDI-II: Beck Depression Inventory - Second Edition ; BAI: Beck Anxiety Inventory; PSQI: Pittsburg Sleep Quality Index; UPDRS: Unified Parkinson’s Disease Rating Scale

	BZD (+)	BZD (-)		P-value
Neurocognitive Measures				
MMSE	29 (26.75-29)	28 (27-29)		0.811
COWAT FAS/PTM	44 (39.5-57)	47 (40-58)		0.961
JLO	51.7 (41.1-55.8)	45 (37.1-50.2)		0.016*
CVLT-2 SDRF	45 (36.3-45)	46.7 (40-55)		0.03*
CVLT-2 LDRF	45 (40-50)	45 (40-50)		0.392
CVLT-2 1-5	44.5 (40.25-52)	46 (38-51.5)		0.349
Trails B	47 (41-57)	49 (43-57)		0.713
Mood and Sleep Self-Reports				
BDI-II	13 (7.5-18)	10 (5-15)		0.046*
BAI	14 (7.5-27)	11 (7-18)		0.039*
PSQI	10 (7-13)	7 (3.5-9.5)		0.039*
Motor Measures				
UPDRS OFF	41 (37-53)	43 (37.25-50)		0.726
# of patients who reported falls	6	15		0.127

Other neurocognitive tests included a measure of global cognitive ability (MMSE: BZD+: 29 (26.75-29), BZD-: 28 (27-29), p=0.811), language ability (COWAT: BZD+: 44 (39.5-57), BZD-: 47 (40-58), p=0.961), long-term memory (CVLT-2 LDFR: BZD+: 45 (40-50), BZD-: 45 (40-50), p=0.392), learning (CVLT-2 1-5: BZD+: 44.5 (40.25-52), BZD-: 46 (38-51.5) p=0.349), and executive function (Trails B: BZD+: 47 (41-57), BZD-: 49 (43-57), p=0.713). Motor measures included the UPDRS when the patients were off PD medications (UPDRS: BZD+: 41 (37-53), BZD-: 43 (37.25-50) p=0.726), and number of reported falls (X2 (1, N=84)=2.33, p=0.127). None of these reached statisticaly significance.

## Discussion

BZDs are widely prescribed for anxiety and sleep disturbances in the general non-PD population [4-6]. Their use however is controversial, as they are linked to adverse effects including increased risk of falls and impaired cognition [7-8]. Given the high prevalence of self-reported anxious mood and sleep dysfunction in PD, BZDs are often prescribed as the first line of treatment for these patients [11-13]. Given the vulnerability of PD patients to develop motor dysfunction and cognitive disturbance due to their underlying disease, the primary aim of the present study was to examine whether PD patients taking BZDs developed further adverse effects compared to PD patients not using these sedatives. 

The data obtained from our sample both support and refute previous studies. With regard to cognition, we found that short-term memory difficulties were significantly higher in the BZD+ group, a finding consistent with the literature [5-7]. On the other hand, in our sample, long-term recall was not significantly different between those taking BZDs and those not, indicating that initial group differences in short-delay recall may be linked to issues with inefficient processing, short-term attention, or consolidation, and not necessarily a longer lasting memory impairment. This has been recognized in the literature as BZDs are primarily believed to cause retrograde amnesia due to their effects on short-term memory consolidation [27].

Further, our finding that patients on BZDs scored significantly better on the Judgement of Line Orientation Test (JLO) is interesting in light of the fact that visuospatial impairments are common in PD patients [19]. To our knowledge, BZD use and better visuospatial function has never been reported in the literature. Whether this particular finding will hold up in future studies remains to be seen, but nonetheless underscores the concept that BZD use is not universally associated with impaired cognitive performance and may be limited to a highly select domain (i.e., short-term memory).

Regarding mood and sleep quality, clinically, BZD+ patients were in the mild-moderate range on the depression scale, moderate-severe range on the anxiety scale, and reported worsened sleep quality compared to BZD- patients who scored in the minimal to mild range for depression, mild-moderate range for anxiety, and reported fair sleep quality. This is important as all of the patients in the BZD+ group were prescribed these drugs in order to better manage their mood and/or sleep disturbances. While taking BZDs did not completely ameliorate mood symptoms and sleep problems, the statistically significant differences between groups reflected very mild symptomology.

Further, research studies have consistently shown that BZD use has been linked to symptoms such as lightheadedness, grogginess, and higher probability of falling [8,9]. Interestingly in our sample, the group not taking BZDs actually fell more and exhibited more motor dysfunction, as measured by the UPDRS, than those taking BZDs. More specifically, 16.67% of patients taking BZDs (6/36) reported falling, in contrast to 31.25% of patients not taking any BZDs (15/48). While this finding may be due to hesitancy from the providers to prescribe BZDs to patients with a higher fall risk, these data serve to reinforce our overall finding that BZD use does not appear to add additional motor compromise in this population.

This study has some strengths and limitations. A significant strength is that this is one of the first studies to examine the effects of BZD use on cognition using a comprehensive neuropsychological battery and motor function specifically in the PD patient population. To our knowledge, there has not been a study investigating adverse effects of BZDs in PD patients in over 40 years. Another major strength is that patients were carefully screened and placed on therapeutic dosages of BZDs under the supervision of a neurologist, while most studies on sedatives include wide ranges of doses, medication use spanning several decades, and patient samples with comorbidities such as psychosis, alcohol, and drug abuse which could inherently alter neurocognitive, motor, mood, and sleep scores [6]. Our study also included a predominantly Hispanic multicultural sample, of which most were Spanish-speaking monolingual, which has not been previously studied regarding BZD use and provides generalizability of our results.

A primary limitation of our study is not correcting for multiple comparisons in our statistical analysis. Given the multiple comparisons, if we apply the appropriate Sidak correction, all of the significant differences disappear. This finding raises an interesting question regarding how to interpret the lack of significance. It is possible that the statistically significant findings before applying the Sidak correction regarding short-term memory, mood, and sleep may represent false positives, which seems unlikely given previous reports in the literature [4-7]. Alternatively, the overall lack of significance after controlling for multiple comparison may speak to the very mild sequelae of BZD use in PD patients. Specifically, we believe this finding further reinforces our argument that BZD use in PD may not necessarily be linked to additional cognitive, emotional, or motor decline.

A second limitation was our small sample size. The current study should be regarded as a first step in exploring the effects of BZD use in PD. Larger sample sizes are needed to achieve the power required to examine a wider range of variables. The influence of BZDs on other mood symptoms, lifestyle variables, and activities of daily living remains unknown.

Another important limitation includes that we did not control for the effects of polypharmacy in our sample, which could possibly also lead to altered cognitive and/or motor function scores. The participants in our study were taking different BZDs at differing dosages at the time of testing. Thus, while we can comment on the overall possible side effects of BZDs as a class, we cannot speak to the specific side effects of any particular BZD or to the safety of dosages above those which we studied. Commonly, adult patients with sleep or anxiety disorders are given up to 4 mg of clonazepam per day to alleviate their symptoms [28]. In our sample, patients ranged from taking 0.5 mg to 5.2 mg of clonazepam equivalents. Therefore, while our findings are most applicable to adult patients receiving typical doses of BZDs for anxiety and sleep disturbances, they might not be generalizable to patients receiving doses outside of this range. 

Despite these limitations, however, our study provides a starting point for understanding the impact of prescribing BZDs for PD patients. An important next step is to focus on dose-dependent relationships between BZDs and mood, motor, and cognitive symptoms in order to further understand how to best manage the range of motor and non-motor symptoms that impact quality of life in PD.

## Conclusions

The present study suggests that the use of BZDs may be associated with select short-term memory changes, but no additional cognitive, motor, or mood dysfunction in PD patients. This study has attempted to fill a gap in data that has lasted 40 years. Our results suggest that though physicians should still be cautious when prescribing BZDs in a PD population, in cases where people are not responding to other medications, benzodiazepines in select patients, may represent a viable alternative. Future research exploring the effects of specific BZDs, dosages, and treatment regimens are warranted for optimal clinical care and to reduce potential for adverse events.
